# A community engagement approach for an integrated early childhood development intervention: a case study of an urban informal settlement with Kenyans and embedded refugees

**DOI:** 10.1186/s12889-022-13185-x

**Published:** 2022-04-11

**Authors:** Margaret Kabue, Amina Abubakar, Derrick Ssewanyana, Vibian Angwenyi, Joyce Marangu, Eunice Njoroge, Eunice Ombech, Mercy Moraa Mokaya, Emmanuel Kepha Obulemire, Catherine Mugo, Tina Malti, Greg Moran, Marie-Claude Martin, Kerrie Proulx, Kofi Marfo, Linlin Zhang, Stephen Lye

**Affiliations:** 1 Institute for Human Development, Aga Khan University, Nairobi, Kenya; 2 Alliance for Human Development, Lunenfeld-Tanenbaum Research Institute, Toronto, Canada; 3 Ministry of Health-Kenya, Nairobi Metropolitan Services, Dagoretti Sub-county, Nairobi, Kenya; 4 Centre for Child Development, Mental Health, and Policy, University of Toronto Mississauga, Toronto, Canada; 5 Department of Psychology University of Toronto, Toronto, Canada; 6 Academics without Borders, Toronto, Canada; 7 School of Psychology, Capital Normal University, Beijing, China

**Keywords:** Community engagement, Informal settlements, Early childhood development, Intervention research, Community health volunteers

## Abstract

**Background:**

Community engagement is crucial for the design and implementation of community-based early childhood development (ECD) programmes. This paper aims to share key components and learnings of a community engagement process for an integrated ECD intervention. The lessons shared are drawn from a case study of urban informal settlement with embedded refugees in Nairobi, Kenya.

**Methods:**

We conducted three stakeholder meetings with representatives from the Ministry of Health at County and Sub-County, actors in the ECD sector, and United Nations agency in refugee management, a transect walk across five villages (Ngando, Muslim, Congo, Riruta and Kivumbini); and, six debrief meetings by staff from the implementing organization. The specific steps and key activities undertaken, the challenges faced and benefits accrued from the community engagement process are highlighted drawing from the implementation team’s perspective.

**Results:**

Context relevant, well-planned community engagement approaches can be integrated into the five broad components of stakeholder engagement, formative research, identification of local resources, integration into local lives, and shared control/leadership with the local community. These can yield meaningful stakeholder buy-in, community support and trust, which are crucial for enabling ECD programme sustainability.

**Conclusion:**

Our experiences underscore that intervention research on ECD programmes in urban informal settlements requires a well-planned and custom-tailored community engagement model that is sensitive to the needs of each sub-group within the community to avoid unintentionally leaving anyone out.

## Background

Urbanization in most low-and middle-income countries (LMICs) is characterized by an upsurge of informal settlements, which are often home to more than half of urban dwellers [1]. Children and families in urban informal settlements are often negatively affected by extreme poverty, overcrowding, unsafe water and poor sanitation, substandard housing, and limited access to basic health and education services [2]. Globally, a high burden of child mortality (close to 3.1 million deaths annually) has also been linked to malnutrition [3]; yet malnutrition remains a common problem in these settlements [4, 5]. These and other factors increase the risk of sub-optimal life-long health and development among children in informal settlements that, in the longer term, have negative implications for human capital development [6–9]. Thus, urban informal settlements pose multiple risk factors for poor health and livelihood, which require timely and responsive interventions.

Community engagement refers to the deliberate integration of communities in designing and implementing research and programme activities, to involve them and their advocates as partners rather than merely research subjects, or eventual users of the intervention [10]. It is crucial for enabling the sustainable implementation of social, economic, and public health research and interventions within urban informal settlements [11–13]. Structures for enabling community engagement can exist independently of the research project, such as through local leaders and community groups, or as structures specifically established by the research organization, such as community advisory boards [13]. Community engagement helps the researcher identify the appropriate structures to promote buy-in, develop mutual trust, and most importantly, sensitize the community on study intentions and seek their support [12]. It also gives researchers a deeper understanding of the contextual factors necessary to develop strategies likely to solicit community involvement and support to the study, while identifying and minimizing internal and external risks [13]. This can demonstrate respect and helps in maximizing benefits for communities. Indeed, positive impact on a range of health and social outcomes, such as health behavior, participants’ self-efficacy, trust, knowledge and attitudes, upgrade of services in slums, and social support, have been linked to community engagement [11, 13, 14]. Reports of unsuccessful research efforts, including the abandonment of trials of tenofovir pre-exposure prophylaxis against HIV infection in Cameroon and Cambodia, have been associated with inadequate community engagement [15, 16]. Community engagement can be impeded by numerous factors, such as funding challenges, the struggle to strike a balance between research and service delivery, complex and contrasting interests among key actors, the requirement for lengthy commitment, overlapping roles, and power dynamics [12, 13]. A poor understanding of the impact of local context, or cultural and ethnic differences, challenges the ability to develop acceptable and meaningful actions to local communities [17].

Few studies have deliberately examined community engagement processes, especially among hard-to-reach communities in urban informal settlements [18, 19]. Available studies do not provide an elaborate description of what processes were successful, and how barriers were overcome. Consequently, little guidance is available regarding the challenge of community engagement in urban informal settlements can be exacerbated further by the presence of various vulnerable sub-populations, such as: immigrants who may lack legal documentation of their residential or nationality status; the presence of multiple layers of leadership and representation owing to the diverse sub-communities; deprivation and exclusion from important services; and insecurity [11, 20, 21]. The distinctiveness of each setting necessitates an inclusive and respectful tailor-made approach that must be informed by a thorough understanding of the local channels of communication and influence to know how best to effectively communicate research or intervention intentions [14]. Moreover, opinion leaders and gatekeepers must be involved to provide the community with a sense of familiarity, ownership and security, and establish the basis for mutual trust [11, 22]. The strong commitment of resources (time, financial and human) and willingness to engage in dialogue while working with the community are crucial [11, 23]. Successful community engagement may require enhancing the skills of researchers who may lack proper training in effective stakeholder engagement processes, due to the absence of a framework to support community engagement within many academic and research institutions [24]. There is existing evidence on the importance of stakeholder engagement process in research [25–27], however, there has been little documented field experiences especially for research involving hard-to-reach populations [19, 28].

Varying degrees of community engagement approaches have been suggested in literature [28, 29] with utilitarian health system perspective and social justice being the overarching two perspectives. In the utilitarian approach the community is are invited when most of decisions on designing, implementation and monitoring of community interventions and research have been made and they may come in to facilitate achievement of certain outcomes, important to the lead implementer. On the flip side, the social justice approach is in form and spirit geared towards community empowerment and ownership, hence keen to engage the community in the process of identification of needs, planning for, and taking part in the implementation and evaluation [29]. Borrowing from previous reports on best practices of community-based participatory research [12], community engagement was chosen as an important first step in implementing an integrated early childhood development (ECD) intervention study in Dagoretti sub-county in Nairobi city. Community engagement was conducted to address three objectives: (i) to identify the appropriate community-level structures to engage the study participants and communities; (ii) to sensitize stakeholders on study intentions and foster a conducive working relationship with them; and, (iii) to enhance the sustainability of the project by soliciting the support of stakeholders, participants, and communities. Although there are no well-established standards, the existing community engagement frameworks and documented studies propose some common broad strategies involving: (i) stakeholder and authority engagement; (ii) formative research; (iii) integration into the local community; (iv) identifying local resources for capacity development; and, (v) shared control and leadership with the local community [19, 22, 30].

In this paper, we present a descriptive summary of activities and experiences of the community engagement process used in the delivery of an integrated ECD intervention study in the urban informal settlement of Dagoretti sub-county. We use the five strategies mentioned above, to describe these community engagement processes by highlighting specific steps and key activities undertaken, challenges faced and benefits accrued from the process drawing from the implementation team’s perspective. We anticipate that the lessons from this case study will generate evidence to support effective and scalable ECD interventions for the most disadvantaged sub-populations in urban informal settlements in LMICs.

### Preliminary work leading to the current study

Formative research was conducted to inform the community engagement and ECD intervention implementation processes. The formative research involved a systematic review of parenting interventions on stimulation and responsive caregiving for children under age 2 years in low- and middle-income countries [31]. Furthermore, between May and June 2018, a household survey was conducted among 458 Kenyan and 118 immigrant households on nurturing care among caregivers of children aged 0–2 years in Dagoretti’s informal settlements [32], and a qualitative study involving 14 focus group discussions with Kenyan and refugee caregivers on ECD practices and experiences [33].

The findings from the systematic review indicated that parenting interventions, which encourage nurturing care effectively improve children’s cognitive, language, motor, and social-emotional development. Moreover, these interventions are most beneficial when delivered in group sessions or group sessions combined with home visits, and are also feasible and effective when delivered by trained paraprofessionals [31]. These findings indicate that parenting interventions with nurturing care components are feasible for the promotion of early child development in low-income settings. Insights from this systematic review were helpful in the justification of the intervention option and, more importantly, informed the intervention study process. The findings from the review are detailed in a separate publication [31].

The household survey captured data on household socio-demographic characteristics, reproductive health outcomes, child health outcomes including vaccination and management of common illnesses, infant and young child feeding practices, activities that promote play, learning and school readiness and childcare and protection practices. The findings of the survey are reported in a separate publication [32]. Broadly, the survey indicated that families with caregivers of low education status, immigrant households, and those with young caregivers were more likely to face greater vulnerability [32]. Child health outcomes were sub-optimal, including: full immunization coverage; infant and young child feeding; child stimulation; and involvement in early-learning activities [32]. These results suggested a need for integrated ECD interventions that are contextually appropriate. The findings of the survey succinctly pointed to the specific gaps and considerations for the planned intervention study.

The qualitative research involved a variety of participants, such as mothers and fathers of young children, community health volunteers (CHVs), refugee caregivers, professionals within the ECD workforce, and it focused on views surrounding ECD practices, experiences, perceived barriers, and facilitators of optimal care for young children [33]. The findings from this qualitative work generally indicated that important ECD gaps and needs include nutrition, economic empowerment, limited time for caregiving, and inadequate involvement of fathers in child caregiving. Some challenges unique to refugees were also identified, including a loss of cultural identity, challenges with legal documentation, which for example limited their accessibility to financial instruments such as MPESA - a commonly utilized digital money transfer platform, thereby impacting negatively on their wellbeing and livelihood. Another emerging finding showed that CHVs were the preferred community-based delivery agent to be considered during the implementation of the integrated ECD intervention.

To further understand the situation on the ground, the study team undertook a situational analysis that identified the social and health services available in the study site, including health facilities (private and public), ECD programmes by other partners, administrative units and local leadership. The study used snowball referral methods to identify services and facilities within the study site. We found that the majority of healthcare providers in the study site were private operators including faith-based health facilities. We also learnt that access to educational facilities in the area was still a key challenge and only two secondary schools are available. The activity provided insights for planning of subsequent activities such as identification of potential sources of partnerships for project sustainability, key informants on various project aspects, potential referral service points for study participants during intervention, and to abate certain unforeseen risks such as duplication of activities/research by other partners.

## Methods

### Study setting

This case study is part of an on-going intervention research being implemented in urban informal settlements of Dagoretti sub-county in Nairobi, Kenya. It targets children between 0 and 2 years and their caregivers from both refugee and Kenyan nationality who reside in Dagoretti. Dagoretti is one of the 17 sub-counties within Nairobi Metropolitan Service region, with most of the area consisting of informal and peri-urban settings. Most of the refugees come from the Great Lakes region, the Horn of Africa, and the Democratic Republic of Congo and moved primarily due to armed conflicts in their home countries. Dagoretti sub-county covers 29 km^2^ and accounts for 10% (approximately 434,208 people) of Nairobi’s population in 2019 [34]. This study focuses on urban informal settlements, which comprise a dense and mixed population of mostly urban poor dwellers who migrate from rural-to-urban areas or other neighboring countries [20]. Like other urban informal settlements in Nairobi, the living conditions in Dagoretti are poor, with many crowded shacks, limited access to piped water, deficient sewage systems, and high crime rates due to unemployment [35].

#### The planned integrated ECD intervention study project

The integrated project is an on-going four-year research study designed to cover the formative and ECD intervention development, the ECD intervention delivery, and evaluation. The main objectives of the intervention study are to identify, translate, adapt, and pre-pilot a set of ECD interventions for use in informal settlements within Dagoretti; and to evaluate the acceptability and feasibility of implementing an integrated ECD intervention in this context. Children less than 2 years and their caregivers from both refugee and Kenyan households form a particular focus of the study. The planned research and intervention activities under the integrated ECD project are reported in a separate study Trial registration: Retrospectively registered in Pan African clinical trial registry on date 26 Mar-2021 (Trial registration number: PACTR202103514565914) [36]. Noteworthy, the insights presented here are drawn from formative and intervention development phases since the remaining phases of the project are pending completion.

### Refugees in the study site

Refugees living in Dagoretti’s informal settlements live within the same residential areas as the host community and have access to shared amenities. Refugees can access most essential social services, including health and education, from the same sources as Kenyans, although in general, services in the informal settlements remain less accessible in comparison to the non-informal settings of Nairobi [35]. In 2014, the government of Kenya issued a directive stating that all refugees living outside an encampment area must relocate to one of the country’s refugee camps [37]. Hence, most of the refugees currently living in the informal settlement lack essential legal identification documents and therefore tend to fear victimization whenever they are involved in community engagement activities, including research. The low numbers and fear of victimization on refugees in the informal settings make them a hard-to-reach study population, risking them becoming invisible.

### Data sources

Data for the current case study is derived from three key sources; i) consultative meetings with ECD stakeholders, government officials and refugee management in Kenya; ii) information from a transect walk undertaken within the community; and, iii) debrief meetings with staff involved in the research implementation. We conducted three consultative meetings with various stakeholders: Kenya’s Ministry of Health working at the Nairobi Metropolitan Health Services and Dagoretti Sub-county; representatives from the United Nations High Commissioner for Refugees (UNHCR), United Nations Children’s Fund (Regional and Country offices), Ministry of Education, African Population Health Research Center and the Aga Khan Foundation- East Africa. In these meetings, we were also accompanied by representatives from the Daraja Civic Initiative Forum; a community-based organization, familiar with the informal settlements studied. These meetings focused on introducing the Ministry of Health officials to the research objectives, solicit authorization to carry out research in the study site, and highlight other support needed (such as community mobilization and planning towards the research execution. Detailed notes of the deliberations during each of these meetings were taken by two staff from the implementing institution. These consultative meetings took place between March and June 2018.

In June 2018, a transect walk across 5 villages (Ngando, Muslim, Congo, Riruta and Kivumbini) within the study site was conducted by three research staff and three representative from Daraja Civic Initiative Forum, guided by a community health assistance and two refugee representatives. During the transect walk, data was collected by two field staff who took observational notes, capturing the available social and health services in the study area.

Lastly, the study team conducted six bi-monthly debrief meetings where we discussed and reflected upon community engagement activities, with an aim to identify what works well and identify areas for improvement. All deliberations from these meetings were documented through taking detailed notes. In this current study, notes from the six debrief meetings held between May and August 2018 were utilized as data sources.

### Data analysis

Detailed notes captured from the stakeholders’ meetings, transect walk, and bi-monthly debrief meetings were scrutinized by three authors (MK, VA and EN). This was followed by a series of discussions with the research team to reach consensus on key themes concerning: how the community engagement was done, reflections on what worked well or needs improvement, and how this informed the ECD implementation study. There was overall consensus for the majority of issues discussed collectively except for the approach and engagement of refugee representatives in our study. The research team initially proposed to engage refugee leaders to provide support through targeted identification and mobilization of refugee-caregivers for the study, and as potential delivery agents of the ECD intervention. To address the lack of consensus about this issue, consultations with ministry of health officials in the study site provided alternative suggestions of considering engaging refugee representatives who are women, since they often live with and interact closely with caregivers of young children in their respective communities. After deliberations it was agreed to pair a refugee woman representative and a Kenyan CHV from the same village, train them, and work jointly in delivering the ECD intervention.

Findings discussed in this paper were synthesized and interpreted with guidance from the commonly documented components of community engagement processes, namely: stakeholder and authority engagement; formative research; integration into the local community; identifying of local resources for capacity development; and shared control and leadership with the local community [19, 22, 30].

## Findings

### Stakeholder engagement

Stakeholder and authority engagement entailed identification, consultation, and sensitization of suitable stakeholders. This was done to ensure that they understood the project structure and goals, and were in a position to negotiate details of the study implementation and more likely to lend their support to the research agenda. As a first step, the study team engaged agencies/institutions carrying out ECD and Health-related work in the regions to identify the players and understand the dynamics involved in the delivery of these to Kenyan nationals and refugees within Dagoretti’s informal settlements. To facilitate this process, face-to-face meetings were conducted with representatives from agencies and research institutions including the United Nations High Commissioner for Refugees (UNHCR), United Nations Children’s Fund (Regional and Country offices), Ministry of Education, African Population Health Research Center, and Aga Khan Foundation (East Africa). UNHCR shared data and resources on refugee populations within Nairobi, although these figures were only indicative and not conclusive because this population is hard-to-reach and under-represented in studies [38]. A common lesson from these engagement meetings was a clear call to work with the existing government departments, especially the Ministry of Health and Community Based Organizations (CBOs), familiar with the study site. Another lesson from these meetings was that as this intervention targeted refugees, there was a strong need to include the host community so as to avoid instigating conflict within the communities. An important opportunity that resulted from these meetings was the sharing of contact information of refugee representatives, as this proved an essential step towards our entry into the community with an embedded refugee sub-population.

The second step involved a series of introductory meetings between the study team and County Health Management Team (CHMT). The CHMT is a body that manages community health at the county, within the devolved government structure under the Ministry of Health. This contact satisfies a formal requirement of the research approving body in Kenya – the National Commission of Science, Technology and Innovation (NACOSTI) [39] - to report to relevant government offices before the commencement of any research activities. This meeting with the CHMT helped introduce the project team to the county officials, communicate the planned research objectives and activities, and solicit their feedback. The CHMT feedback helped in refining the planned community entry processes, research objectives, design and study implementation strategies. The CHMT also provided authorization to work in the study site and introduced the project team to Dagoretti sub-County Health Management Team (SCHMT); a health management team at the level of the Sub-County (i.e. a lower level from the CHMT). The SCHMT is better integrated into the community and has established structures and networks from the household level up to the policy-makers level.

The third step in stakeholder engagement was to meet the SCHMT with similar objectives as those in meetings with the CHMT. The meeting was also aimed at collectively establishing a community mobilization strategy. A critically important benefit of this meeting was the introduction of the project team to the Community Health Volunteers (CHVs) and their supervisors. CHVs are community members who provide health-related services in their communities, with some formal but limited training provided by the health system or health program which sponsors their work [40]. The work of the CHVs addresses critical shortages in the health workforce and strengthens primary healthcare systems targeting global health goals [41]. The engagement of the research team, SCHMT and the CHVs formed a strong partnership that has continued to facilitate collaboration and smooth running of the research activities in this study setting. Overall, throughout this process, we learned that the key stakeholders relevant for our intervention include; the Ministry of Health, community health structures (CHMT and SCHMT, CHVs), community-based service organizations working in the study site, local leaders, and refugee leaders.

### Formative research

Formative research aims to obtain a deeper understanding of the local context, identify particular needs, and map key stakeholders, contributing to the final study design [19]. For this study’s formative research component, we conducted a systematic review, situational analysis and household survey all described in the background section of this case-study paper. Overall, findings from formative research provided the study team with extensive information on the following aspects; population distribution, social and health needs and service delivery infrastructure; the terrain of the study site; socioeconomic status; social determinants of child health and development; the existing governance structures and key stakeholders working with the study community. This information was crucial for guiding the intervention design, selecting evaluation measures, and implementing a contextually appropriate and integrated ECD intervention. The formative work also helped the study team build a relationship with the community leaders and CHVs.

### Identification of local resources for capacity development

Identifying local resources for capacity development refers to the mapping of community resources whose capacity can be enhanced to advance the agenda of the study. This might include human resources such as Community Health Volunteers (CHVs) and other service providers targeted for skill development, and physical resources such as training and meeting venues within the community. Based on the observations made during the transect walk across the study site, the study team discovered that some parts of the study site were nearly impassable, especially during the rainy season and that this would necessitate proper logistical considerations during project activities such as data collection and community meetings. We also learned that refugees in the study site were embedded within the host native communities and they both shared same community infrastructures like water sources, health facilities and schools. This activity also generally provided important insights into the general needs and services within the study setting. The activity was also important for planning subsequent activities by enabling better identification of potential sources of partnerships for project sustainability, sources of information (such as key informants on various project aspects), and potential referral points for study participants during an intervention. This activity also highlighted certain unforeseen risks, such as duplication of activities/research by other partners.

Furthermore, as part of identification of resources, the research team identified the Ministry of Health’s community structure, which has a well-established network of CHVs who deliver health and social services to households in the study site as key for this research. During the deliberations with stakeholders, we learned that it is beneficial to work with the CHVs for a number of reasons which include: there is already established community trust of the CHVs, there is clear assignment of a specific number of households to each CHV, there is an established monitoring and supervision plan for CHVs by the MOH, and that basic training is given to CHVs on service delivery, disease prevention and surveillance. However, during our engagement with CHVs, we recognized that this approach (i.e. solely relying on CHVs) was not sufficient, especially for reaching out to the refugee/immigrant sub-populations as these were hard-to-reach. We learned that refugee leaders are those most trusted by their communities. However, following deliberation with ministry of health officials in the study site, we ended up working with refugee women as opposed to refugee leaders. The refugee leaders had challenges in committing ample time, the majority of them were male and lacked the lived experience of day-to-day engagement with caregivers of young children. Thus, we recruited refugee women to work with the study team to plan, identify, and mobilize the refugee/immigrant study participants. It is noteworthy that this engagement with refugee women revealed that, despite their remarkable influence on community action, they lacked capacity in health services delivery compared with their CHV counterparts. Training the refugee women was therefore incorporated in the intervention implementation plan to ensure that they effectively reach the refugee sub-population.

Furthermore, the implementing organization employed some people from the local community to be part of the study team, that is, in the capacity of enumerators and community mobilizers. Residents with basic skills in community development activities were identified, recruited and trained. This helped in navigating the study site, since this team was familiar with the contextual facilitators and barriers such as terrain and culture and they helped advise on the strategies to use.

### Integration into local lives

Integration into local lives entails aligning the research activities with the community’s daily activities and common practices and, thus, minimizing disruptions of the daily routine [19]. Towards this end, our study team used numerous meetings to integrate itself within the community-level health structure to ensure community buy-in, and build the necessary relationships. Meetings were usually attended by the Ministry of Health fraternity representing the different health programmes and stakeholders in health service delivery. Other meetings include those by CHAs with the CHVs representing different parts of the community, where they report quarterly progress and discuss the challenges they face in the field. The meetings provided an opportunity to continually learn about the health and social dynamics within the study site, including service utilization, specific health needs of the community, upcoming activities either by partners or the government, existing opportunities, and sources of weaknesses or risks for our planned intervention study. This also provided the team with a platform to share study related information and findings on the on-going research work. One of the negotiation outcomes was also that our project would sometimes host such meetings to support the Ministry of Health agenda and integrate into the community.

Furthermore, we learnt from the transect walk, household survey and situational analysis that the majority of informal settlement dwellers depended on inconsistent daily wages in employment that demanded extra effort, often featuring walking long distances, long hours of work, and non-uniform patterns or work shifts. Our assessment from this experience was that any intervention activities that would interfere with study participant’s employment/working arrangements would be received with much resistance, and would hinder the smooth progress of the research project. We, therefore, ensured that there was meaningful and ample involvement of the caregivers (as study participants), refugee leaders and CHVs (as delivery agents) in the design of the research activities. The input and consultation with participants and delivery agents ensured that the community engagement and research activities were well integrated into local structure and schedules of the community dwellers to avoid disruptions and resistance from the community. For instance, as a result of other demands on caregiving and livelihood activities, caregivers preferred to be engaged in study activities during midmorning hours and for not more than 2 h per day. Moreover, the project team considered the need to compensate transport expenses and provide an allowance for the time spent by the study participants while taking part in the study activities since their other activities of livelihood and income generation would be inconvenienced.

To further integrate into the local lives of the community, the long-term employment arrangement of the community members throughout the study period also meant that the skills acquired from training and the hands-on experience were sustained within the study team and could be drawn on for future or new project activities compared to circumstances where project employees are short-term consultants. Moreover, the employment of community members as part of the project team is expected to initiate and nurture a trusting relationship between the study community and the project team.

The project also established a research office within the study site to enhance improved integration into the community. The office was established as a contact point by the study participants and/or community whenever they had any issues or consultations regarding the study. The office serves as a venue for planning meetings and for training activities. The research office also houses some assessment facilities where data collection activities such as neurodevelopmental assessments are conducted. These would otherwise be difficult to execute in households due to challenges such as confined spaces and lack of confidentiality.

### Shared control and leadership with the local community

Shared control or leadership involves building the capacity of community members to take on responsibilities and actively participate in decision-making regarding research study implementation and transferring responsibility accompanied by support and supervision [30]. Communities’ willingness to participate in interventions significantly depends on the extent to which organizations are willing and able to share control [42]. Our community engagement practices ensured that the selected CHVs and refugee leaders/representatives were meaningfully involved in planning and mobilization processes, during which they exercised some level of leadership and control over the project activities. This process started by orienting and seeking their input on the research objectives, processes, and expected outcomes. The study team further described the potential benefits that the community could expect from the research activities in the area. These deliberations were followed by a series of consultative meetings on the study implementation strategy. These meetings involved the study team, CHVs and refugee leaders who represented the community’s interests. While the study team had already generated a sampling strategy and data collection procedures, engaging with the CHVs and refugee leaders helped to identify contextual issues that could stand in the way of research. For instance, it was apparent that data collectors should not directly approach households because they were not well known and might therefore generate resistance or security issues. It was consequently decided during consultation meetings that data collectors be accompanied by CHVs or refugee leaders/representatives to introduce them to the participating households and provide security as they are known and trusted within their areas of jurisdiction. This process also exposed the CHVs and refugee leaders to research processes thereby improving their capacity to serve the community.

Involving CHVs and community leaders in planning and executing study activities is also expected to contribute to their empowerment and enhance ownership and trust from the community. These are crucial aspects for promoting the sustainability of the research and intervention outcomes. Besides, shared control and leadership with the community members such as the CHVs and refugee leaders addresses the research team’s workload yet concurrently builds the capacity of both community members and the research team, which is a mutual benefit for both parties.

## Discussion

Lessons from the current case study indicate that the involvement of Ministry of Health leadership and related community health structures (CHMT, SCHMT, CHVs), community-based service organizations working in the study site, local leaders, and representatives of the target group throughout the planning and implementation of integrated ECD interventions is critical. Proper stakeholder engagement promotes an alignment of healthcare research with the needs of service providers, beneficiaries, and policymakers [43].

Noteworthy, implementation research on ECD for populations in urban informal settlements with embedded refugees requires a well-planned community engagement process that is sensitive to the unique needs, available resources, and structures of the communities involved. These findings corroborate with literature on public engagement process tenets that highlight the value of engaging known agents from the local community and building relationships of trust [11]. As such, settings with vulnerable and marginalized sub-populations such as refugees require meaningful representation and involvement of all the different groups. Their fear of victimization, language barrier, and lack of legal identification documents are potential barriers that could easily render this community invisible in research. Underrepresentation of populations in research can result into poor service planning and delivery, challenges in quality improvement, as well social injustice [44]. Disadvantaged or marginalized populations may often be occupied with earning a living and thus inclined to pay less attention to healthcare, science, or research [19, 45–47]. In addition, such populations may be overwhelmed by the demands of daily life that pose tremendous challenges in the delivery of any parenting interventions [48]. To some communities, their cultural experiences and historical events may become the main source of resistance to interventions from perceived “outsiders” [13, 19]. One key solution to reach these populations is to integrate engagement activities into their daily lives and common practices [19]. Therefore, researchers, programme implementers and policymakers ought to be sensitive to these barriers and adopt community engagement approaches that are tailored to their unique needs.

We highlight that formative research plays the complementary roles of feeding into the community engagement planning process and ensuring that the process is tailored to specific needs and the realities within the communities. Indeed, researchers in the fields of ECD emphasize that it is important to conduct formative data collection prior to developing an integrated intervention as this facilitates the process of contextualizing the intervention approaches, messages and materials to maximize the opportunities for behavior change [49]. Formative research expands on the local contextual knowledge prior to, and as part of, a community engagement effort and to enhance the understanding of dynamics of influence and communication [14]. Formative research can help to identify logistical barriers and increase the chances of program acceptability and effectiveness [50]. This noted, the lack of guidelines on how to conduct formative research for integrated research and interventions is still problematic [49]. Our experience shows that the use of mixed research methods, which involve a variation in the respondent representation (e.g. caregivers, program staff, health workers, etc.), as well as triangulation between qualitative and quantitative research, provides rich data which can help in identifying unforeseeable challenges and crucial needs to address within the community.

The need to build local capacity coupled with the need to integrate into the local lives are strong pillars for building trust and securing project sustainability. As lesson from the current case study, working with existing community structures should involve shared control of leadership roles to ensure that the community members’ capacity is built, that they are empowered, respected, and take ownership of the project or actions that seek to bring the improvement in their communities. Indeed, a growing body of research emphasizes the need to invest in training, equipping, retention and motivation of ECD workforce, but also importantly, the need to adapt sustainable approaches towards ensuring that the capacity of people from local communities is enhanced so that they are in a position to promote ECD outcomes in their communities even though programs come to an end [51, 52]. Besides, building local capacity is crucial for enhancing participants’ involvement in ongoing research and plays an important role in project sustainability and engagement in future interventions [53].

We learned that as much as CHVs appointed within ministry of health structure are well respected and recognized in the general community, their reach and influence within the embedded refugee sub-population can be complex and at times hampered by various challenges including language barrier, low level of trust, among other issues. This finding has useful implications for planning and implementing ECD interventions in communities with potentially marginalized sub-groups such as immigrants, as they may miss out on services which are presumed to be within their reach. We recommend the need for engaging refugee representatives and building their capacity on ECD, and that there is great benefit in pairing CHVs and trained refugee representatives to work as a team for cross-learning and experience sharing.

Our findings indicated that there is a myriad of competing priorities coupled with underlying social determinants of health which can become serious impediments to involvement and access of ECD interventions within an urban informal settlement. Similar competing interests like domestic chores, search for food, transportation challenges and other social determinants like gender inequalities have been identified in other research on ECD programs [54, 55]. There is need for cognizance of competing interests and dynamics for participation and uptake of ECD and thereby the need to incorporate new understandings of culture-based perceptions about ECD, and to improvise different modalities and communications of ECD during the planning of ECD interventions [56].

The model adopted in this study addressed most of the community engagement needs for the research study underway. However, the model did not address the challenges that come with highly mobile refugee population and urban informal settlement dwellers; especially considering that this implementation research study was designed to run for more than 3 years. This meant that some of the community members engaged early in the study at different levels migrated to other areas within the country or abroad and there were new ones who came to live within the project site. In the course of the study, three refugee representatives have moved from Kenya to other countries; which meant that new refugee representatives had to be engaged. Likewise, seven Kenyan study participants relocated from the study site to other areas in Nairobi. The refugee representatives moved to other countries during the 4 months’ period that the study implementation team retreated to develop the integrated intervention, that is, after the formative research phase. The implication was that we had to do a fresh recruitment of other refugee representatives to help with planning, mobilization and implementation of the intervention; an aspect that did not only derail the process but also caused anxiety in regards to the viability of involving them among the implementation team. The lesson we learnt from this is that when carrying out research among mobile populations such as refugees and dwellers in the urban informal settlement, there should be flexibility in terms of timelines and resources to address emerging issues. Furthermore, we also learned from our community engagement model, that although it helped the study implementation team to appropriately engage the ministry of health and community structures, there was still uncertainty on how to best to engage other stakeholders, especially those from the Civil Society and private sectors within the study site.

The community engagement model used in this case study has borrowed heavily from utilitarian health system perspective underpinning the level of community engagement [29]. Through the formative research, we gathered information on the needs in the community, designed an intervention and engaged CHW and refugee representatives to deliver thereby leveraging their credibility, empathy and contextual awareness. Elements of social justice approach are demonstrated by the empowerment of delivery Agents (drawn from the community) with skills and knowledge of administering an ECD integrated intervention, as well as the trickle-down effect to the caregivers. This continues to help them achieve better outcomes for children during the intervention period and beyond. The limitation with this approach is the potential risk of dependency by the community members in designing interventions to address their needs due to the lack of complete involvement in; setting agenda, identifying priorities, choice of research design and package of the intervention. Besides, the study’s limited timelines and resources available would not have allowed for use of the social justice approach which is associated with community empowerment, shared control and leadership. Our current study only describes initial stages leading to the implementation of an integrated ECD intervention, yet community engagement is an ongoing process. Our community engagement process did not reach fruition within this limited timeline, and therefore more lessons on integration into local lives and shared control and leadership will emerge during the actual implementation of the ECD intervention. As more experiences about the specific needs, services, health and social and cultural dynamics within the community are picked by the implementation team, the process of suitably aligning the programme activities and longer term goals with numerous realities within the community will evolve organically.

### Study strengths and limitations

A major strength of this study is its focus on community engagement within an under-researched urban sub-population (i.e. informal urban settlement), which helps to generate new lessons/knowledge on conducting sustainable research and interventions under situations of complex power structures, resource constraint, cultural diversity and other dynamic factors. Our work also demonstrates how community engagement can be used to inform the specific components of implementation research (e.g. recruitment process, duration/scheduling of research activities, intervention delivery agents, capacity needs etc) and vice-versa. This noted, our findings should be interpreted with caution for various reasons. First, it is difficult to generalize the findings from this case study since the conclusions are only about the participants being observed and the conclusions are based in implementation team’s perspectives. Besides, the forms of approach and level of success of community engagement strategies described in our study are likely to be different among communities with different refugee compositions, and varying community level resources and structures. As an example, the stakeholders’ engagement process was limited to the requirements and expectations of Nairobi county government which is potentially different from other counties considering the autonomy of the devolved governments in Kenya.

Future research is needed on community engagement processes in urban marginalized and hard-to-reach communities similar to our settings for better generalizability. Also, future research should explore the longer-term benefits of community engagement beyond the project duration. Besides, the steps described under the community engagement process in the current case study were used in the pre-COVID era and thus may not be sufficient for the current realities of COVID-19 pandemic. During the pre-COVID-19 era, most community engagement activities relied on face-to-face interactions, whereas during COVID-19, various forms of restriction necessitated an increased use of alternative interactive engagement approaches, such as virtual meetings, virtual trainings and telephone-based communication. More research is however required to identify further aspects and modifications which can ensure adequate community engagement for ECD implementation in the era of COVD-19, similar pandemics and other forms of disaster. Lastly, this study draws conclusions based on perspectives from a study implementation team for the ECD integrated intervention, which can be subjective and potentially biased.

## Conclusions

The current case study is a step-by-step account of a community engagement process for an integrated ECD intervention within an urban informal settlement that is a hard-to-reach setting with diverse sub-populations. We demonstrate that a well-planned and custom-tailored community engagement model that is sensitive to the needs of each sub-group, including native and migrant population, within the community is of paramount importance, while efforts should be made to that none of the communities and sub-groups are unintentionally left out. To achieve such impactful community engagement may require context relevant strategies such as timely stakeholder engagement, undertaking meaningful formative research (e.g., through desk reviews, transect walks, household surveys and in-depth interviews with local community members), identification and support/building local resource capacities and enabling shared control or leadership, as well as meaningful integration into the local lives.

## Data Availability

The datasets used and/or analyzed during the current study are available from the corresponding author on reasonable request.
